# Chylopericardium after cardiac surgery can be treated successfully by oral dietary manipulation: a case report

**DOI:** 10.1186/1749-8090-4-44

**Published:** 2009-08-18

**Authors:** Sing Yang Soon, Sharath Hosmane, Paul Waterworth

**Affiliations:** 1 South Manchester University Hospital NHS Trust, Southmoor Road, Manchester, M23 9LT, UK

## Abstract

We report a case of chylopericardium after ascending aorta and aortic valve replacement, which presented as late tamponade. We discuss the various treatment options in this rare condition which can result in serious morbidity or death.

## Introduction

Chylopericardium after intra-thoracic surgery is rare. Its incidence is reported to be between 0.22% to 0.5% [1,2] following paediatric cardiac surgery but is not quantified following cardiac surgery in the adult population. A delay in diagnosis can lead to serious consequences with tamponade and death [3]. Chronic lymph leak can also lead to immunosuppresion, hypoproteinemia and malnutrition [3]. The majority of published literatures on this condition after cardiac surgery are in children. There are few reports of chylopericarium in adults following coronary artery bypass surgery and valvular surgery [4-6], and these advocate treatment with either total parenteral nutrition or surgical intervention. We report on the first case of chylopericardium after ascending aorta and aortic valve replacement in an adult patient treated successfully by oral dietary manipulation.

## Case report

A 52 years old man who presented with an incidental finding of an aortic regurgitant murmur underwent further investigations which reveal a dilated ascending aorta (5.1 cm at its widest point) and associated aortic regurgitation. There was no other significant past medical history. He subsequently underwent aortic valve replacement with a mechanical prosthesis and also ascending aorta replacement with a PTFE interposition tube graft. The thymic fat was divided in the midline. Cardiopulmonary bypass was established with a single two-stage venous cannula and aortic return was to left femoral artery. There was no intra-operative complication and the patient made an uneventful post-operative recovery. He was discharged on the 8^th ^post-operative day at which time he was well and a chest x-ray did not show any signs of cardiomegaly.

**Figure 1:**
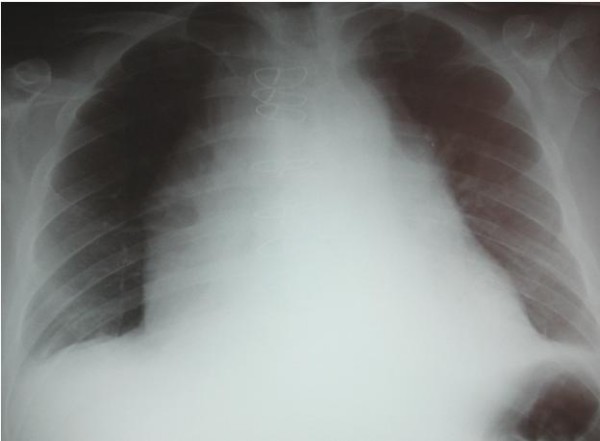
**CXR showing patients enlarged mediastinal shadowing from chylopericardium**.

The patient represented on the 12^th ^post operative day with increasing shortness of breath, accompanied by nausea and vomiting. Chest x-ray showed gross cardiomegaly (fig 1). Echocardiography demonstrated a 6.5 cm pericardial effusion with diastolic right ventricular collapse. A pericardial pigtail catheter was inserted for relief of tamponade with drainage of 3.0 litres of milky white fluid. Subsequent biochemical and microbiological analysis confirmed sterile chyle.

Due to presence of the prosthetic aortic valve and Dacron graft, our aim was to avoid total parenteral nutrition with its attendant risk of prosthetic infection. Therefore, a decision was undertaken to treat the chylopericardium by a trial of oral dietary manipulation with medium chain triglycerides/fat free diet. The second day after pericardiocentesis was performed, the drainage was still substantial at 1.5 litres. However on the third day, the drainage tailed dramatically to 150 ml. The patient was brought to theatre for creation of a subxiphoid pericardial window with insertion of 32F drain for more effective drainage. The chyle leak continued to diminish in volume over the next five days, without any drainage by day eight. However on application of low pressure (10 cm of water) suction on day nine, a small piece of debri was dislodged from the drain and there was a sudden drainage of 450 ml of chyle. Therefore thrice daily low pressure suction was instituted.

The patient spiked a temperature the following day to 39.5 degree Celsius. A full septic screen was performed including blood cultures and the chyle was sent for microbiological analysis. Initially, the patient was commenced on broad spectrum antibiotics. Subsequently, gram negative bacilli were found to be growing in both the blood cultures and the chyle. Treatment with meropenem was instituted. The patient responded to the antibiotics treatment and became apyrexial after seven days.

By day 20 post readmission, the drainage had tailed off to less than 20 ml per day. The patient was subsequently commenced on a normal diet. The drain output was observed closely for 5 days after reinstitution of normal diet. There was no further chyle leak. An echocardiogram confirmed no re-accumulation in the pericardial sac and the drain was therefore removed and the patient discharged. The white cell, lymphocyte and albumin count remained within normal limits throughout the patient's readmission even during the septic episode.

## Discussion

Chylopericardium after cardiac surgery is rare and therefore a high index of suspicion is required for its diagnosis. Its aetiology is usually due to disruptions of the tributaries of the thoracic duct rather than to the main duct itself [2]. The thoracic duct originates as the cisterna chili adjacent to the second lumbar vertebrae. It ascends anterior to the vertebral bodies and enters the thorax through the aortic hiatus. It is a predominantly right sided structure and crosses over to the left at the level of the fourth and fifth thoracic vertebrae. It empties the lymph that it transports into the left jugulosubclavian venous junction. It has a highly variable intra-thoracic course. There are also various tributaries found in the pericardial reflections and thymic tissues that confluences to the thoracic duct [1,7]. Therefore, one should ensure that division through the thymic tissues and pericardium be conducted carefully to prevent subsequent chyle leakage. Other causative factors include caval obstruction, subclavian vein thrombosis, congenital lymphangiectasia, filariosis and medistinal tumors [8].

Chyle leak is suspected with the appearance of milky effluent in the chest drain. Confirmation comes with biochemical analysis of the fluid that reveals presence of chylomicrons, cholesterol, lactate dehydrogenase and protein [8-10]. Cytology usually demonstrates a lymphocytic picture while microbiological culture is invariably sterile.

Upon diagnosis, there are various treatment options available. Although nutritional support with parenteral hyperalimentation has been advocated as the method of choice [6,9,11], we advocate one of minimal intervention with a dual strategy of decreasing lymph production and ensuring adequate protein intake to counter any effects of the potential hypoproteineamia. As first line management, the patient should be commenced on a trial of enteral nutrition with a fat free diet or a low-fat diet with medium chain triglycerides, which are absorbed directly into the portal system rather than through the lymphatics. This would reduce the production of lymph and allow the spontaneous closure of the fistula in the majority of cases. This option is also more palatable for the patient and avoids the potential complications of total parenteral nutrition. It also has the added theoretical benefit of promoting normal gut flora and preventing translocation of pathogens in a patient that might be leukopenic. Care must also be given to ensure that the patient has adequate caloric and nitrogen intake in a highly catabolic state. Pericardial decompression should be achieved with either a pig-tail catheter inserted under echocardiography or the insertion of a drain with the creation of a subxiphoid window

The duration of treatment is variable, but typically lasts for 7 to 21 days [1,7,12]. Regular monitoring of the albumin and leukocyte count should be carried out to assess the nutritional and immunological status during the length of enteral/parenteral treatment. Consistent fall of both of these counts are relative indications for operative intervention should the chyle drainage be small (less than 500 ml/day) yet persistent. Cessation of chyle drainage usually indicates successful treatment. However, it is prudent to request a repeat echocardiography to assess pericardial effusion prior to drain removal in the event of drain blockage with debri.

If the above measures do not result in the resolution of the chyle leak, operative intervention needs to be considered. There is no clear consensus about indications for surgery but it has been recommended that if chyle drainage is greater than 500 ml per day for 5 consecutive days or failure of conservative treatment after 14 days or if metabolic complications developed [7,13,14].

The identification of the site of chyle leak can be problematic. A lymphangiogram can be performed preoperatively to give an indication of the area where the leakage is situated. Other measures to assist in the location of chyle leak include asking the patient to consume methylene blue or a high fat cream one hour prior to the surgery [9]. The area involved would stain blue or exude thick milky fat at the time of operation.

After localizing the culprit lesion, ligaclips or simple ligatures could be employed to deal with the problem. Problems arise when one fail to localize the site of drainage. Mass ligature of the thymic tissues and diathermy of the pericardial reflection should be carried out on a "best guess" basis. Plication of all the tissues anterior to the vertebral bodies from the level of the azygous vein to the level of the proximal descending aorta has also been advocated [8]. Other options of intervention include right sided video assisted thoracoscopic ligation of the thoracic duct [7] which has been reported to be without any rate of recurrence at four years.

In conclusion, patients with chylopericardium after cardiac surgery can potentially be treated effectively with oral dietary manipulation with a medium chain triglyceride diet and effective pericardial decompression. This approach would reduce the complications associated with total parenteral nutrition and the attendant morbidities of surgical interventions. Monitoring of the rate of chyle leakage will guide subsequent therapy.

## Consent

Written informed consent was obtained from the patient for publication of this case report and accompanying images. A copy of the written consent is available for review by the Editor-in-Chief of this journal.

## Competing interests

The authors declare that they have no competing interests.

## Authors' contributions

SYS – Manuscript writeup, SH – Carried out image scanning, patient consent, manuscript upload and revision. PW – Senior author, provided guidance and input on manuscript writeup. All authors read and approved the final manuscript.
